# Editorial: Translational Research and Drug Repurposing for Non-Communicable Diseases (NCDs)

**DOI:** 10.3389/fphar.2022.879611

**Published:** 2022-03-21

**Authors:** Abidemi J. Akindele, Amos A. Fatokun, Ikemefuna C. Uzochukwu, Vanessa Steenkamp, Christian Agyare, Oluwole Familoni, Margaret O. James

**Affiliations:** ^1^ Department of Pharmacology, Therapeutics and Toxicology, Faculty of Basic Medical Sciences, College of Medicine, University of Lagos, Lagos, Nigeria; ^2^ Centre for Natural Products Discovery (CNPD), School of Pharmacy and Biomolecular Sciences, Liverpool John Moores University, Liverpool, United Kingdom; ^3^ Department of Pharmaceutical and Medicinal Chemistry, Faculty of Pharmaceutical Sciences, Nnamdi Azikiwe University, Awka, Nigeria; ^4^ Department of Pharmacology, Faculty of Health Sciences, University of Pretoria, Pretoria, South Africa; ^5^ Department of Pharmaceutics, Faculty of Pharmacy and Pharmaceutical Sciences, Kwame Nkrumah University of Science and Technology, Kumasi, Ghana; ^6^ Department of Chemistry, Faculty of Science, University of Lagos, Lagos, Nigeria; ^7^ Department of Medicinal Chemistry, University of Florida, Gainesville, FL, United States

**Keywords:** drug repositioning, cancer, cardiovascular diseases, diabetes, neurodegenerative diseases

## Background

Translational research involves scientific (collaborative) explorations aimed at putting investigative findings in the basic medical sciences into useful applications in clinical practice for the purpose of prevention, diagnosis, and therapeutic management of disease conditions. This “bench to bedside” approach facilitates the emergence of new medications. Drug repurposing (repositioning) is a relatively recent but promising evolution within this context that refers to investigational initiatives to put currently approved drugs, agents previously withdrawn, and relatively out-dated drugs to new therapeutic uses in the clinic. Reported examples of successes in drug repurposing include sildenafil (original indication (OI)—angina; new indication (NI)—erectile dysfunction); thalidomide (OI—morning sickness; NI—erythema nodosum leprosum and multiple myeloma); aspirin (OI—analgesia; NI—colorectal cancer); ketoconazole (OI—fungal infections; NI—Cushing syndrome); and topiramate (OI—epilepsy; NI—obesity). In respect of the ongoing Coronavirus Disease 2019 (COVID-19) pandemic, clinical trials of drugs approved for other indications, including remdesivir, ivermectin, dexamethasone, toclizumab, mavrilimumab, baricitinib, baricitinib with remdesivir, etc., have shown some beneficial effects (Chakraborty et al., 2021).

## Goal

The incidence, morbidity, and mortality associated with non-communicable diseases (NCDs) have been on the increase worldwide over the years. Cardiovascular diseases, chronic respiratory diseases, cancers, diabetes and mental illnesses have been reported to be major contributors to the NCDs burden in developing and developed countries. In most cases, cures are unavailable and current palliative therapeutic interventions are associated with worrisome untoward effects. In fact, it has also been recently shown that NCDs as pre-existing conditions are major contributors to the severity and fatality associated with COVID-19 worldwide. Drug repurposing in translational research offers a great opportunity for widening the therapeutic options for the treatment of NCDs. The advantages over the usual drug discovery and development pipeline include lower associated risks and conservation of time and cost.

## Scope

Our research topic provided a focused platform for researchers in translational and drug repurposing research to share their findings as regards NCDs and to showcase potential leads and alternate therapeutic options towards reducing the burden of NCDs worldwide. The scope included reviews and research articles on *in silico*, *in vitro* and *in vivo* experimentations, or combinations thereof, with potential impact on the prospect of finding novel treatments for NCDs, including cardiovascular diseases, chronic respiratory diseases, cancers, diabetes, mental illnesses, etc.

## Overview of Contributions

Trastuzumab (TZM) is a monoclonal antibody employed in the treatment of human epidermal growth factor receptor 2 (HER2)-positive breast cancer and metastatic gastric cancer (Waller et al., 2021). Cardiac dysfunction is a cause for concern in the use of this biologic. This effect has been linked to the downregulation of neuregulin-1 (NRG-1) which plays a vital role in the activation of cell survival pathways in cardiomyocytes and the maintenance of cardiac function (Zeglinski et al., 2011). Olorundare et al. evaluated the therapeutic potentials of selected antihypertensive medications (amlodipine, lisinopril, valsartan, and their fixed-dose combinations) in TZM-induced cardiotoxicity. Parameters assessed include markers of cardiac injury and oxidative stress, along with profiling of lipids and histopathological and immunohistochemical assessments. The authors reported that treatment with the antihypertensive drugs and their fixed-dose combinations reversed derangement in biochemical parameters, and histopathological and immunohistochemical presentations induced by TZM. The beneficial effect of the interventions was linked to antiapoptotic and oxidative stress inhibition mechanisms. However, cause for concern was raised with valsartan/lisinopril fixed-dose combination in terms of associated hyperlipidemia, increased atherogenic index and coronary artery index values, and coronary artery cartilaginous metaplasia.

Alzheimer’s disease (AD) is a neurodegenerative disorder characterised by initial deficits in the ability to encode and store new memories and later progressive changes in cognition and behaviour, in which symptomatic treatment offers marginal benefit on cognition, but the condition seriously warrants the emergence of disease-modifying therapies (Soria Lopez et al., 2019). The pathophysiology of AD relates to beta-amyloid deposition and neurofibrillary tangles in association with the triggering of a complex cascade of events (leading to neuronal cell death, loss of neuronal synapses and progressive neurotransmitter deficits), sustained immune response and inflammation, and significant prion-invoked increase in abnormal proteins, leading to brain damage (Kinney et al., 2018). Xu et al. *via* enrichment analysis of drug-induced transcriptional profiles of pathways based on AD-associated risk genes identified from genome-wide association analyses (GWAS) and single-cell transcriptomic studies sought to identify potential anti-AD agents. Their study unveiled ellipticine, alsterpaullone, tomelukast, ginkgolide A, chrysin, ouabain, sulindac sulfide and lorglumide as potential anti-AD agents. The authors suggested further experimental validation and that their approach may be applied in repurposing drugs for other neurological disorders.

Hepatocellular carcinoma (HCC) is the most common primary liver malignancy responsible for significant cancer-related mortality worldwide and with increasing numbers in terms of incidence and mortality, despite various advances (Balogh et al., 2016). According to Kirstein and Wirth (2020), interdisciplinary and multimodal treatment strategies are essential for successful therapy of HCC, including surgical interventions, local ablative therapies, locoregional therapies and systemic therapies (sorafenib—multityrosine kinase inhibitor; lenvatinib, regorafenib and cabozantinib—tyrosine kinase inhibitors; ramucirumab—VEGF-receptor inhibitor, etc.). Abd El-Fattah et al. explored the potential of sitagliptin (an antidiabetic drug) for the management of HCC using the N-nitrosodiethylamine-induced HCC mouse model. The authors established that sitagliptin inhibited HIF-1α activation via interference with the AKT-AMPKα-mTOR axis and interruption of IKKβ, P38α and ERK1/2 signals. Sitagliptin demonstrated antiproliferative (inhibition of angiogenesis and stimulation of apoptosis), anti-inflammatory (diminution of TNF-α level and downregulation of MCP-1 gene expression) and antifibrotic (reduction in TGF-β level) effects. Their findings provide justification for the repurposing of sitagliptin for the treatment of HCC.

Alam et al. in a bid to identify drugs that could be repurposed across a range of seemingly unrelated conditions adopted an integrative network approach to delineate genetic overlaps in pathophysiology between tuberculosis (TB)—an infectious disease—and non-communicable diseases (NCDs) such as Parkinson’s disease, cardiovascular disease, diabetes mellitus, rheumatoid arthritis and lung cancer. Through their interaction network of disease genes, authors identified 86 hub genes linked to inflammatory/immune and stress responses as commonly associated with TB and its overlapping NCDs. A network of drugs targeting those genes was then built, and their drug-target interactions were explored. Such drugs or drug combinations targeting the hub proteins are suggested to have potential to improve clinical conditions in co-morbidities involving TB and overlapping NCDs, and could potentiate or synergise with one another to achieve better outcomes. These possibilities represent a promising future application of drug repurposing (drug repositioning).

## Conclusion

The articles published within this research topic capture the unique and exceptional promise of drug repurposing in NCDs, as well as showcasing the multidisciplinary approaches and experimental tools and platforms being deployed to advance drug repurposing research. The collection enables a reader to appreciate how drug repurposing is revolutionising drug discovery and development. While maximising the benefits of drug repurposing certainly requires further integration of basic research and clinical practice (bench-to-bedside and bedside-to-bench), our research topic signifies we could be witnessing a new era in finding relevant drugs in a more sustainable manner—at least, with respect to time and speed—to manage several disease conditions.
